# The Associations between Perceived Parenting Styles, Empathy, and Altruistic Choices in Economic Games: A Study of Chinese Children

**DOI:** 10.3389/fpsyg.2017.01843

**Published:** 2017-10-16

**Authors:** Qingke Guo, Linlin Feng

**Affiliations:** Department of Psychology, Shandong Normal University, Jinan, China

**Keywords:** empathy, perceived parenting styles, altruistic behavior, other-regarding preferences, Chinese children

## Abstract

Parenting styles are critical for fostering children’s empathy and prosociality. Yet these relations haven’t been well established for Chinese children, and the underlying mechanisms were seldom explored. Drawing upon parental acceptance-rejection theory and empathy-altruism hypothesis, the objective of this study was to investigate the relationships between perceived parenting styles and altruistic behavior of children, and the intervening role of children’s empathy and the moderating role of in-group and out-group conditions. What is novel about this study is that it contains both survey data and experimental data. Four hundred and ninety-four children (*M*_age_ = 8.92 years) completed four simple binary-choice dictator games which are widely used in the study of other-regarding preferences (concerns for the interests of others). These children also reported their perceived parenting styles. And children’s empathy was reported by their mothers. Each child’s altruism score, which was used in the subsequent analyses, was derived from the altruistic choices in these games. Mediation analyses indicated that, when age and gender were controlled for, maternal and paternal emotional warmth were positively associated with children’s altruism via children’s empathy, while maternal and paternal rejection were negatively associated with children’s altruism via children’s empathy. Multi-group analyses showed that the influences of perceived parenting styles on children’s altruistic behavior via children’s empathy were consistent for in-group and out-group conditions. These findings suggest that enhancing parental emotional warmth and reducing parental rejection may foster children’s empathy, which in turn promote children’s altruism. Limitations and future directions of this study were also discussed.

## Introduction

Humans often behave altruistically, care about the well-being of others, worry about unfair distributions of wealth, reciprocate kind or unkind behaviors of others, and so on. These behaviors can be labeled as prosocial behaviors. Among these behaviors, altruism can be defined as intentional and voluntary behaviors that people act to increase other people’s welfare as their ultimate goal rather than as merely a means to an end designed to enhance the actors’ welfare (Batson et al., 2002). Rather than relying on the traditional methods of questionnaires or observations, a growing number of studies use economic games or allocation games to measure these behaviors. Using the experimental paradigm of other-regarding preferences (e.g., altruism, fairness, inequality aversion), several researchers (e.g., Fehr et al., 2008, 2013; Bauer et al., 2014) have made fruitful contributions. In their experiments, a considerable number of children were regarded as altruists since they always choose the allocations that maximize the partner’s payoff. Altruism has also been found to vary with age, gender, in-group and out-group conditions, and so on (Fehr et al., 2008, 2013; Bauer et al., 2014).

The motivational source of altruistic behavior is probably empathy (Eisenberg et al., 2006). As the empathy-altruism hypothesis has posited, the prosocial motivation aroused by empathy can be targeted at improving the welfare of others especially those in need as the ultimate goal (Batson et al., 1991). Empathetic children are better attuned to the emotional state of others. This sharing of emotional state can effectively promote prosocial behaviors, as well as inhibit harmful behaviors (Eisenberg et al., 2006; Miller et al., 2014). Empirical researches also suggest that parenting styles may be important in fostering children’s empathy (Motta et al., 2006; Kanat-Maymon and Assor, 2010) and altruistic behavior (Padilla-Walker and Christensen, 2011). Therefore, it is reasonable to postulate that the influences of perceived parenting styles on children’s altruism may be intervened by children’s empathy.

To date, there has been a scarcity of literature exploring the influence of parenting styles on children’s altruism in the use of experimental economics paradigms, particularly in Chinese background. Therefore, the primary aim of the current study was to explore both positive and negative parenting styles that can foster or reduce children’s altruism, as well as the intervening role of children’s empathy and moderating role of in-group and out-group conditions. Simple economic games (e.g., dictator games) can provide an avenue for systematically examining altruistic behavior even for young children. Therefore, in this study four simple binary-choice dictator games (Fehr et al., 2008, 2013; Bauer et al., 2014) were adopted as objective measures of children’s altruistic behavior. These economic games can effectively eliminate the influence of social desirability and improve the ecological validity of the results.

### Perceived Parenting Styles, Children’s Empathy, and Altruistic Behavior

Family is the first socializing context to foster children’s prosocial behaviors as well as inhibit their antisocial behaviors. In accordance with socialization theorists (e.g., Hoffman, 2000), parents are important for fostering their children’s prosocial behaviors. A large body of literature has demonstrated that, a supportive parent-child relationship characterized by mutual positive affect and cooperation is beneficial for children’s prosocial development (e.g., Malti et al., 2013; Grusec and Hastings, 2015). Parental warmth refers to the parenting style characterized by positive affect, responsiveness, and support for children (Carlo et al., 2010). Previous findings have suggested several ways that parental warmth and positive involvement promote children’s prosociality and emotional responsiveness to others. First, nurturing parents offer a model of parental caring and comforting behaviors as well as emotional concern that is likely to be emulated by their children (Grusec and Hastings, 2015). Second, parental warmth and involvement can enhance children’s feelings of belonging to collectivity and connectedness with others by fostering the sense of security, trust, and protection (Hoffman, 2000). Moreover, warm and supportive parents are beneficial in encouraging children to understand their own and others’ emotions and needs, as well as regulate their emotions (Malti et al., 2013). All these features may promote children’s prosociality.

On the other hand, negative parenting styles such as rejection and overprotection have been suggested to be detrimental to children’ altruism, by deterring the development of children’s empathy and social skills (Fosse and Holen, 2002; Romero-Martínez et al., 2013). For example, parental rejection during childhood may be responsible for the development of violence and the lack of adequate empathy in adulthood (Romero-Martínez et al., 2013). Furthermore, parental overprotection may inhibit children from developing adequate social skills for dealing with problematic contexts (Fosse and Holen, 2002).

However, most of the aforementioned researches were conducted in Western countries. To date, there is still a scarcity of literature focusing on the relationships between perceived parenting styles and children’s prosocial development in Chinese culture. We only found one study conducted in mainland China, which focused on investigating the influences of parenting styles on 3- to 5-year-old children’s social inhibition and voluntary helping in experimental situations (Liu and Yang, 2004). However, the researchers did not pay attention to different roles of mothers and fathers as well as the intervening mechanism.

Empathy refers to the affective responses that result from comprehension of others’ emotional state, which involve better understanding of others’ emotions and sharing of these emotions (Taylor et al., 2013). Considerable empirical researches have corroborated the claim that parenting styles may be important in fostering or destructing children’s empathy. Social cognitive theorists have speculated that warm parenting styles can foster and model empathy (e.g., Eisenberg, 1986). According to the parental acceptance-rejection theory (Rohner, 2004), parental behavior was a bipolar dimension with parental acceptance standing at one pole while rejection standing at the other. Strayer and Roberts (2004) have tested a model that socialization of empathy involves both parents’ and children’s factors. Parents’ factors such as parental emotional warmth, may positively predict children’s empathy, while parental rejection may do the opposite.

Empirically, positive parenting practices were found to be beneficial to the development of empathy in elementary school children (Motta et al., 2006), while negative parenting practices perceived by the children were found to be detrimental to the children’s inclination to respond empathically to others (Kanat-Maymon and Assor, 2010). Though the importance of empathy in promoting children’s social development has been strongly stressed by Western researchers, literature focusing on parental roles in children’s empathy development is still limited in China. Western cultures are often characterized by individualism and personal agency, while Chinese culture is often characterized by interdependence and mutual connection (Li et al., 2013). In China, strong parental control was usually viewed as an expression of love (Chao, 1994). As a result, maybe parental overprotection in China had less negative effects on Children’s moral development than in Western backgrounds.

Previous literature neglected the roles of fathers in influencing children’s empathy and altruistic behavior. In Chinese society, mothers are the primary caregivers for children, suggesting that mothers may exert greater influences on children’s development than fathers. For example, one study in China has found that maternal parenting stress and parental psychological aggression would have effects on children’s problem behavior, while paternal behavior would not (Liu and Wang, 2015). However, with the social and economic development in China, more and more mothers have full-time job and fathers may act as coparents rather than helpers. It is reasonable to expect that paternal behavior is also important for children’s development as fathers in China are more actively engaged in child care.

### The Importance of Children’s Empathy in Prosocial Development

Empathy has been considered as a key motivator for altruism. In consistent with the empathy-altruism hypothesis (Batson et al., 1991), Carlo et al. (2011) found that empathy, including empathic concern and perspective-taking, was positively related to prosocial behaviors of Mexican American college students, including helping in anonymous, emotional, dire, compliant, and public situations as well as altruism. Klimecki et al. (2016) found that empathy can increase altruistic behavior in dictator games by comparing an empathy induction condition and a control condition. There is also evidence that empathetic concern was the key predictor of prosocial behaviors among older adolescents in Hong Kong (Siu et al., 2012). These results may cross-culturally verify the importance of empathy in motivating altruism (Batson et al., 1991).

The intervening role of empathy in the relationships between perceived parenting styles and children’s altruism has been suggested by relevant findings. Krevans and Gibbs (1996) discovered that the associations between parents’ discipline and children’s prosocial behaviors were intervened by children’s empathy. Specifically, children with inductive parents were found to be more empathic, and more empathic children were found to be more prosocial. But Krevans and Gibbs (1996) have not distinguished the different roles of maternal and paternal parenting. Padilla-Walker and Christensen (2011) also confirmed the intervening role of empathy between parenting and prosocial behaviors toward strangers and friends, yet only positive parenting styles (including parental involvement and parent–child connectedness) were examined. Taylor et al. (2013) suggested that mothers’ emotion socialization was correlated with the development of children’s empathy and was benefit to their later prosocial behaviors with peers, but they did not explore the influences of fathers. Besides, previous studies on these relations were mostly conducted with Western samples. Thus the associations between perceived parenting styles and children’s altruism as well as the potential role of children’s empathy as an intervening variable in Chinese culture were nearly neglected.

### The Moderating Role of In-group and Out-group Conditions

The development of parochialism (i.e., in-group favoritism and the out-group hostility) in children has received research interest only in recent years. Fehr et al. (2013) found that parochialism developed significantly in the teenage years. Specifically, the teenagers were more willing to share their resources with in-group members than with out-group members, and were more spiteful toward out-group members than toward in-group members. Benozio and Diesendruck (2015) found that parochialism emerges at a much younger age than that proposed by Fehr et al. (2013). For example, 3- to 6-year-old boys (but not girls) allocated more resources to in-group versus out-group recipients, and protected common goods when they were alone. Padilla-Walker et al. (2016) also found that the teenagers reported more prosocial behaviors toward in-groups (family and friends) than toward out-groups (strangers). Further, they also provided indirect evidence that parenting styles may have different effects on prosocial behaviors toward multiple targets. Specifically, maternal warmth was associated with children’s prosocial behaviors toward family, while paternal warmth was associated with prosocial behaviors toward friends. Moreover, maternal hostility was not associated with prosocial behaviors toward any target while paternal hostility was negatively associated with prosocial behaviors toward family, friends, and strangers. Based on these findings, we propose that the effects of perceived parenting styles on children’s altruism via children’s empathy may differ for in-group and out-group conditions.

### The Present Study

Childhood may be an important period for the development of empathy and prosocial tendencies that emerge during earlier stages. Existing literature has studied the developmental trajectories of other-regarding preferences in children (Fehr et al., 2008, 2013; Bauer et al., 2014), yet little progress has been made whether they are affected by parenting styles and empathy during childhood.

Nowadays, due to China’s One-Child Policy, many Chinese children face a special situation that they do not have siblings to interact with (Chen et al., 2013). Previous researchers worried that these only children would be spoilt by their parents and become self-centered “little emperors” (Cameron et al., 2013). Thus Chinese children’s prosocial behaviors and the influencing factors should be particularly paid attention to.

As far as we know, this may be the first paper that adopted both experimental and correlational methodology to study the link between perceived parenting styles and altruism as well as the mechanisms in Chinese background. Since the father’s roles in rearing children are becoming increasingly important, the influences of both maternal and paternal parenting styles were analyzed in this study, respectively. Moreover, this study focused on not only positive parenting styles such as emotional warmth, but also negative parenting styles such as rejection and overprotection. This study was aiming to investigate which perceived parenting styles are beneficial to children’s empathy and altruism, and which perceived parenting styles are detrimental.

In this study, the prospective associations between perceived parenting styles, children’s empathy and children’s altruism throughout childhood (7–11 years) were examined. The central goals were twofold. The first goal was to examine the influences of perceived parenting styles on children’s empathy and altruistic choices in economic games. The second goal was to explore the moderating effect of in-group and out-group conditions. Children are expected to become more altruistic, less selfish, and less likely to be spiteful (i.e., a tendency to minimize the partner’s payoff) with increasing age (Fehr et al., 2013; Bauer et al., 2014). Girls are supposed to be more empathetic and more prosocial than boys (Barr and Higgins-d’Alessandro, 2010; Siu et al., 2012). Thus both the aforementioned investigations have been controlled for the effects of age and gender.

In this study, parental emotional warmth was hypothesized to be positively correlated with children’s empathy and altruism, while rejection would be negatively associated with children’s empathy and altruism. Overprotection may not be associated with children’s empathy and altruism. Moreover, it was hypothesized that perceived parenting styles would significantly influence children’s empathy, which in turn significantly influences children’s altruism. Additionally, in-group and out-group conditions were expected to moderate the associations between perceived parenting styles and children’s altruism via children’s empathy.

## Materials and Methods

### Participants and Procedure

The data was collected during one assessment in 2015. Ethical approval for the present study was obtained beforehand from the Institutional Review Board at Shandong Normal University. Recruitment occurred via parent-teacher meetings followed by consent forms and measures being submitted to parents. Parents who agreed to participate to this study returned the completed consent forms and questionnaires (including a demographics section and the Griffith Empathy Measure), while parents who did not agree to participate in this study and their children were not included in this study. Children were made aware of the voluntary and confidential nature of this research before the assessment and were also asked to sign consent forms. In this study, 525 children whose parents had agreed to participate were asked whether they would like to play, 504 children agreed to participate. There were 21 children who did not suit up for participation because they were not feeling well the day of testing. Specifically, 525 children were initially invited to participate, and all their parents agreed the children to participate. Children were asked to complete the measure of their perceived parenting styles, with all items read by trained research assistants with elaborate explanations to ensure the children’s fully understanding and appropriate responses. Then the children completed four simple binary-choice dictator games.

Fehr et al. (2013) have implemented a design that was supposed to be able to avoid socially desirable behaviors in individual interactions among participants and experiments. We adopted this experiment design and applied it to all age groups. We also ran the experiments in class during regular school hours with pen and paper. Specifically, each time about 20–25 children in one class played the games, with nobody being able to see what the children sitting nearby did. Each participant was informed that his/her partner (an imaginary participant) was also playing this game simultaneously. Before making choices, the children should answer a series of questions about the payoff meanings of each option correctly to ensure their comprehension, and 10 children who failed to do so were excluded from further analyses. At last, a total of 494 children (241 boys, 253 girls, *M*_age_ = 8.92 years, *SD* = 1.04) from two public elementary schools in Shandong province, Eastern China, were included in this study. The sample was in large part a working and middle class sample. Apart from receiving gifts in return for their participation, the children were also paid according to their choices in these binary-choice dictator games to insure their incentive and concentration (Fehr et al., 2008, 2013; Bauer et al., 2014).

### Measures

#### Altruism

Altruism was measured using four binary-choice dictator games, as suggested by Bauer et al. (2014). Each child was told that there would be four games and he/she was matched with an anonymous partner of the same age. Each child was told that there was a real partner in other classroom, and he/she was paired with the partner randomly. In each game, the participants had to choose between two alternative allocations of stickers for them and for an anonymous partner. The order of presentations of the two allocations and the four games was randomized. Note that, using one-shot experiments with anonymous partners enables the experimenters to distinguish altruistic behavior from selfishly motivated behaviors. If the experiments involve repeated interactions or face-to-face contacts, the participants may behave altruistically in order to benefit from future reciprocity (Fehr et al., 2013).

The costless prosocial game offered a choice between the allocation (1, 1), that is, one sticker for him/herself and one sticker for a partner, and the allocation (1, 0), that is, one sticker for him/herself and no sticker for a partner. This game measures the most basic form of altruism. It is costless to increase the partner’s payoff when the subject chooses (1, 1). The costless envy game offered a choice between the allocation (1, 1) and the allocation (1, 2). In this game, the choice of (1, 2) can increase the partner’s payoff at no cost to the subject, but this choice may result in a disadvantage for the child. The costly prosocial game offered a choice between the allocation (1, 1) and the allocation (2, 0). The choice of (1, 1) implies a costly transfer of a valued resource to the partner. In this game, selfish children are more likely to choose (2, 0) because the provision of a benefit for the partner is costly. The costly envy game offered a choice between the allocation (1, 1) and the allocation (2, 3). In this game, these choices lead to a higher reward for the subject and the partner. The choice of (2, 3) indicates altruism because the subject provides a benefit for the partner (Fehr et al., 2008, 2013; Bauer et al., 2014).

Altruism of the subjects was based on their choices in these four binary-choice dictator games. The children were denoted as altruistic if they selected the altruistic choices in these dictator games, that is, they always assign a positive value to another person’s payoff. In accordance with this, the selection (1, 1) in costless prosocial game, the selection (1, 2) in costless envy game, the selection (1, 1) in costly prosocial game, and the selection (2, 3) in costly envy game were considered as altruistic choices (Bauer et al., 2014). The altruistic choices were coded as 1 point, while the other choices were coded as 0 point. The children’s altruism scores were computed by summing the scores across four games, and higher scores indicate higher levels of altruism (ranging from 0 to 4).

We also performed an in-group condition and an out-group condition in the participants. We assigned half of the participants to the in-group condition and half to the out-group condition randomly. The recipients in the in-group condition were known to be from the same class (the participants were instructed to imagine playing the game with a class-classmate), while the recipients in the out-group condition were known to be from another school (the participants were instructed to imagine playing the game with an anonymous stranger from another school) but were of the same age cohort (Fehr et al., 2013). When finished, the participants took away the stickers they allocated to themselves in these four binary-choice dictator games.

#### Perceived Parenting Styles

Perceived parenting styles were measured with the Chinese version of s-EMBU (the short-form Egna Minnen Beträffande Uppfostran; Jiang et al., 2010). Each item was asked separately regarding the children’s mothers and fathers. The s-EMBU is a 42-item measure including three dimensions: (1) rejection means degrading the children verbally, being hostile and critical, and using punishment (six items; e.g., “My mother/father was sour or angry with me without letting me know the cause.”); (2) emotional warmth means physically and verbally support, loving attention, stimulation, and acceptance (seven items; e.g., “My mother/father praised me.”); (3) overprotection means being worried, controlling for the children, and being anxious for children’s safety, and high expectations for their achievement (eight items; e.g., “I wished my mother/father would worry less about what I was doing.”). While the item “I was allowed to go where I liked without my parents caring too much” was deleted due to its low discrimination. In consideration of the perception of parental behaviors may be more of importance than actual parental behaviors, children’s reports were adopted rather than parents’ reports. Children indicated the frequency with which their mothers/fathers engaged in the practices described on a four-point scale (1 = *No, never*; 4 = *Yes, nearly always*). The scores of the relevant items were taken, with higher scores representing greater rejection, emotional warmth, and overprotection. This measure has been validated in China and show satisfactory reliability and validity (Jiang et al., 2010). In this study, the Cronbach’s alpha coefficients for maternal rejection, emotional warmth, and overprotection were 0.69, 0.75, and 0.50, respectively; for paternal rejection, emotional warmth, and overprotection were 0.74, 0.75, and 0.55, respectively.

#### Empathy

The Chinese version of the Griffith Empathy Measure (GEM; Dadds et al., 2008), including 23-item brief parent-report questions, was adopted in this study. Statements were as follows: “My child becomes sad when other children are sad” and “My child can’t understand why other people get upset.” Given that females take more responsibility in childrearing (Wong et al., 2009) in China, GEM was only administrated to mothers, just as Dadds et al. (2008) did. Each item of GEM was rated on a five-point scale (1 = *strongly disagree*; 5 = *strongly agree*). The total score of GEM was computed by summing up all items, with higher scores reflecting a greater degree of empathy. GEM was found to have appropriate psychometric properties for Chinese population (Zhang et al., 2014). In this study, the Cronbach’s alpha coefficient was 0.76.

### Statistical Analyses

Structural equation modeling (SEM) was utilized to examine the relationships between perceived parenting styles and children’s altruism, as well as the intervening role of children’s empathy and the moderating role of in-group and out-group conditions in this study. SEM analyses were conducted using Amos 17.0 with maximum likelihood estimation. Evaluations of the SEM models were based on these following statistics conventionally: the Comparative Fit Index (CFI), the Tucker-Lewis Index (TLI), and the Root Mean Square Error of Approximation (RMSEA) (Kline, 2005).

## Results

### Descriptive Statistics and Correlations

Following Fehr et al. (2013) who studied how the distribution of altruism score develops with age, we grouped the participants according to their age. Children were grouped into 4 age cohorts in this study, namely 7–8, 8–9, 9–10, and 10–11. For example, children in the age group 7–8 were all elder than 7 but younger than 8. Here 8 was the upper limit of that group. If a child was 8-years-old, he/she belonged to the age group 8–9. So actually there was no overlap in the age groups. The main effects of age and gender and the interaction between the two were assessed with ANOVA. Significant age difference in altruistic choices was found, *F*(3,486) = 14.46, *p* < 0.001, $\eta_{p}^{2}$ = 0.08. Elder students were generally more altruistic than younger students; they made more altruistic choices than younger students, with some exceptions in the age group 8–9 (*M*_7-8 years_ = 1.92, *SD*_7-8 years_ = 1.08; *M*_8-9 years_ = 1.84, *SD*_8-9 years_ = 1.03; *M*_9-10 years_ = 2.39, *SD*_9-10 years_ = 1.25; *M*_10-11 years_ = 2.63, *SD*_10-11 years_ = 1.19). The main effect of gender and the interaction between age and gender were not significant, *F*(1,486) = 0.96, *p* > 0.05, $\eta_{p}^{2}$ = 0.00; *F*(3,486) = 1.51, *p* > 0.05, $\eta_{p}^{2}$ = 0.01.

Means, standard deviations of the key variables used in this study and correlations among them were presented in **Table 1**. Results showed that high parental emotional warmth was related to higher levels of children’s empathy, and high parental rejection was related to lower levels of children’s empathy and altruism, high parental overprotection was correlated with lower levels of children’s altruism. Additionally, children’s empathy was positively correlated with their altruism.

**Table 1:** Correlations among the Key Study Variables.

	1	2	3	4	5	6	7	8	9	10	11	12
(1) Maternal rejection	–											
(2) Maternal emotional warmth	–0.25**	–										
(3) Maternal overprotection	0.39**	0.15**	–									
(4) Paternal rejection	0.63**	–0.19**	0.32**	–								
(5) Paternal emotional warmth	–0.20**	0.77**	0.10*	–0.28**	–							
(6) Paternal overprotection	0.27**	0.12*	0.74**	0.40**	0.17**	–						
(7) Empathy	–0.18**	0.32**	0.03	–0.16**	0.33**	0.07	–					
(8) Costless prosocial	–0.08	0.03	–0.11*	–0.12**	0.05	–0.15**	0.07	–				
(9) Costless envy	–0.04	0.03	–0.02	–0.05	0.01	–0.03	0.07	0.25**	–			
(10) Costly prosocial	–0.14**	0.06	–0.12**	–0.13**	0.06	–0.13**	0.12**	0.33**	0.15**	–		
(11) Costly envy	–0.03	–0.00	–0.01	–0.04	–0.01	–0.04	0.04	0.00	0.30**	0.08	–	
(12) Altruism score	–0.12**	0.07	–0.11*	–0.13**	0.05	–0.13**	0.13**	0.63**	0.68**	0.64**	0.55**	–
*M*	9.51	20.78	14.93	9.40	19.81	13.91	77.14	0.70	0.35	0.45	0.70	2.20
*SD*	3.24	4.44	3.71	3.46	4.57	3.78	11.94	0.46	0.48	0.50	0.46	1.18

*^∗^*p* < 0.05; ^∗∗^*p* < 0.01.*

### Structural Equation Models

**FIGURE 1:**
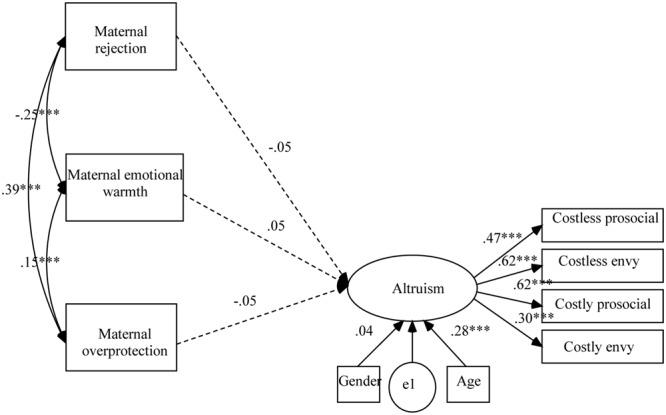
Results of structural equation analysis evaluating the direct effects of maternal parenting styles on children’s altruism. ^∗∗∗^*p* < 0.001.

**FIGURE 2:**
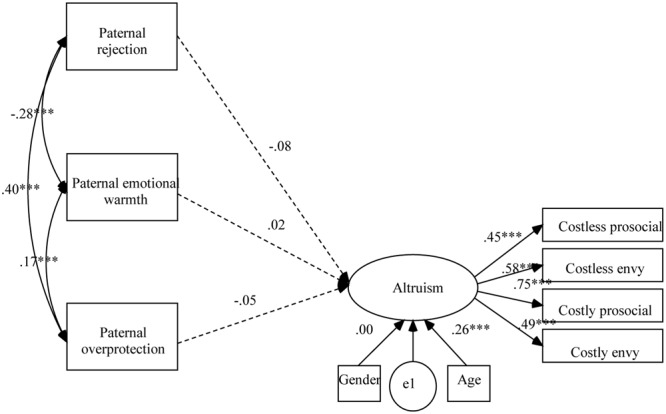
Results of structural equation analysis evaluating the direct effects of paternal parenting styles on children’s altruism. ^∗∗∗^*p* < 0.001.

Based on the previous correlation analyses, the direct effects of maternal and paternal parenting styles on children’s altruism were first evaluated. The models fit the data well, CFIs > 0.96; TLIs > 0.92; RMSEAs < 0.041. But the effects of maternal parenting styles on children’s altruism (as shown in **Figure 1**) and the effects of paternal parenting styles on children’s altruism (as shown in **Figure 2**) were all insignificant.

Although perceived parenting styles do not affect children’s altruism directly, it is still possible that they have indirect effects. According to Holmbeck (1997), intervening effect can be distinguished into mediating effect and indirect effect. That is, when independent variable has no direct effect on dependent variable, it may still produce a significant indirect effect via the intervening variable. In this part, though perceived parenting styles showed no direct effect on children’s altruism, its indirect influences via children’s empathy may still be evident. Therefore we proposed two hypothesized structural models: (a) Maternal parenting styles → children’s empathy → children’s altruism, and (b) Paternal parenting styles → children’s empathy → children’s altruism. The effects of age and gender were controlled, by adding paths from them to the dependent variables.

**FIGURE 3:**
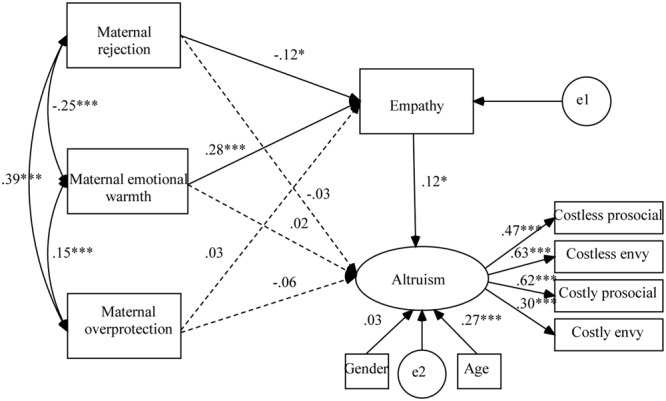
Results of structural equation analysis evaluating the intervening effect of children’s empathy between maternal parenting styles and children’s altruism. ^∗^*p* < 0.05; ^∗∗∗^*p* < 0.001.

**FIGURE 4:**
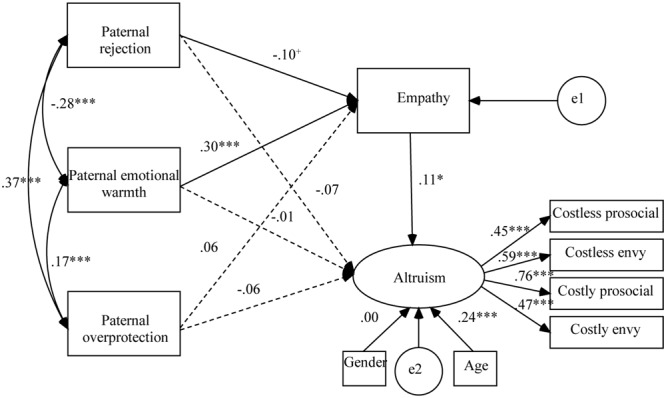
Results of structural equation analysis evaluating the intervening effect of children’s empathy between paternal parenting styles and children’s altruism. ^+^*p* < 0.10; ^∗^*p* < 0.05; ^∗∗∗^*p* < 0.001.

These two hypothesized models fit the data well, CFIs > 0.96; TLIs > 0.92; RMSEAs < 0.038. As shown in **Figure 3**, maternal rejection and emotional warmth had indirect effects on children’s altruism through children’s empathy (*p*s < 0.05). The overall model explained 11.40% of the variance in children’s altruism. In addition, as shown in **Figure 4**, paternal rejection and emotional warmth also had indirect effects on children’s altruism through children’s empathy (*p*s < 0.05). The overall model explained 10.10% of the variance in children’s altruism.

### Multi-Group Analysis

To examine whether in-group and out-group conditions can moderate the intervening models, multi-group analyses were used to compare an unconstrained model (i.e., the structural coefficients were permitted to vary for in-group and out-group conditions) with a constrained model (i.e., the structural coefficients were set equal for in-group and out-group conditions), in accordance with the chi-square differences corresponding to the values of degrees of freedom. As presented in **Table 2**, the chi-square differences between the unconstrained model and constrained model were insignificant for both maternal and paternal models. Thus the hypothesis of moderating effect of in-group and out-group conditions was not supported.

**Table 2:** Goodness-of-Fit Indices for the Multi-Group Structural Equation Models.

	χ^2^	*df*	χ^2^/*df*	CFI	TLI	RMSEA	Δ*df*	Δχ^2^	*p*
**Maternal model**									
Unconstrained	55.536	46	1.207	0.98	0.96	0.021	7	3.202	0.866
Constrained	58.738	53	1.108	0.99	0.98	0.015			
**Paternal model**									
Unconstrained	44.463	42	1.059	1.00	0.99	0.011	7	2.441	0.931
Constrained	46.904	49	0.957	1.00	1.00	0.000			

## Discussion

This study is on a “hot topic”: children’s empathy and prosocial behaviors. The impact of a relatively new factor, perceived parenting styles, on children’s empathy and prosocial behaviors was explored. This study was conducted in the background of Chinese culture; a new-important but nearly neglected cultural context in the literature on the relationships between perceived parenting styles and children’s prosociality. Cameron et al. (2013) documented that China’s One-Child Policy has produced significantly more self-centered and less cooperative individuals. This has led to widespread concern within China about the social skills of these only children. This study distinguished maternal and paternal parenting styles and looked at their effects on children’s prosociality separately. This study had two main goals: First, it was aimed to determine whether empathy intervens the relationships between perceived parenting styles and altruistic choices in economic games. Second, it was aimed to examine whether the relationships are consistent between in-group and out-group conditions.

In the structural equation models, perceived parenting styles showed no direct effects on children’s altruism. This is consistent with Yoo et al. (2013), who proposed that children’s perceptions of “what their parents do” might not be related with whether they behave prosocially toward others. Similarly, Padilla-Walker et al. (2016) also failed to reveal significant direct association between mothers’ warmth and children’s prosocial behavior toward strangers. The absence of the direct effect of perceived parenting styles on children’s prosociality could be interpreted as the results of the development of autonomy and independence during childhood, as well as values and other dispositional characteristics that are directly associated with behaviors (Padilla-Walker et al., 2016).

Given the central importance of empathy in prosocial development (Eisenberg et al., 2006), it is proposed that empathy may be such a role that “carries” the influences of perceived parenting styles on children’s altruism. That is, specific perceived parenting styles (e.g., rejection and emotional warmth) may influence children’s perspective taking and emotional sensitivity, which in turn, may influence altruistic behavior. In other words, in spite of the absence of direct influence, perceived parenting styles may exert indirect influences on children’s altruism through children’s empathy.

Not surprisingly, perceived parenting styles (maternal rejection and emotional warmth, as well as paternal rejection and emotional warmth) were found to relate indirectly to children’s altruism through the effects of children’s empathy. Our findings support the intervening role of empathy in the relations between perceived parenting styles and prosocial behaviors (Padilla-Walker and Christensen, 2011; Taylor et al., 2013), thus confirmed the central importance of empathy in children’s prosocial development (Batson et al., 1991; Eisenberg et al., 2006). Empathic children are more likely to act in altruistic ways because they are sensitive to other individuals’ needs and engender greater concern for the well-being of others (Eisenberg et al., 2006). This study, in concert with recent findings (e.g., Ding and Lu, 2016), has provided support for empathy-altruism hypothesis in Chinese background. It suggests that the mechanism that empathy motivates altruism also applied to collectivistic cultures.

We deduced that emotional warmth was particularly outstanding in fostering children’s empathy and altruism among three types of perceived parenting styles. When perceived parenting style is emotional warmth, children may perceive their parents as trustful and protective, and thus engender sense of security, which may encourage them to understand and share the emotions and needs of their own and of others. Moreover, parental warmth and supports may provide a caring model for children, and increase both children’s willingness to attend to parental messages and the accuracy in detecting these messages (Hoffman, 1970). Additionally, warm and no rejecting parents may share these prosocial traits with their children through shared genes, modeling, and other environmental means. All these are beneficial to the development of children’s empathy and altruism. But when perceived parenting style is rejection, children may think that their parents do not respect their feelings and personal opinions. Thus the children cannot pay attention to others’ needs and engender feelings of caring. As a result, the children cannot interact with peers in respecting and caring. These features are detrimental for the development of empathy and altruism.

Although previous research suggested that parental overprotection in Chinese culture may have less negative effects on children’s prosocial development, compared with that in Western cultures (Fosse and Holen, 2002). Chinese children may have been accustomed to their parents’ protection, a common parenting behavior in China, thus experience less aversions and frustrations (Jiang et al., 2010). However, in the present study, both maternal and paternal overprotection was negatively correlated with children’s altruism. These results suggested that latest developments in urban China might have weakened the influence of traditional cultural notions, as China’s Open Door policy permits influence of Western cultures which emphasize the value of personal independence. In this context, the meanings attached to parental control in contemporary China may be different from those in the past, and children may view their parents’ continual monitoring and behavioral corrections to be more of an intrusion upon their sense of autonomy (Zhao and Wang, 2014).

This study has hypothesized that the influences of perceived parenting styles on children’s empathy and altruistic behavior may differ for in-group and out-group conditions. However, multi-group analyses produced insignificant chi-square differences between the unconstrained model and the constrained model, suggesting that the relations between perceived parenting styles, children’s empathy, and children’s altruism were similar for in-group and out-group conditions.

But when children grow older, the increasing exposure to and membership in social groups may trigger different behaviors for in-group and out-group conditions. To support this, Fehr et al. (2013) found that parochialism (different treatments of in-group and out-group individuals) emerged in adolescence. Specifically, significant in-group favoritism was observed at the age of 14 to 15 years, and significantly stronger spitefulness to out-group individuals was observed from the age of 12 to 13 years. Benozio and Diesendruck (2015) found that parochialism emerges at a much younger age than that proposed by Fehr et al. (2013). For example, 3- to 6-year-old boys (but not girls) allocated more resources to in-group versus out-group recipients, and protected common goods as if they were alone. One explanation is that sensitivity to group membership is particularly important for males in human evolution. Another explanation is socialization, that is, boys are encouraged to “engage in competitive and group-like interactions” (p. 263). Findings in China showed that children’s group consciousness may emerge since grade 2, but unstable. As they grow older, children’s group consciousness become stable and mature until grade 6 (Fu et al., 2002). In the present study, participants were mostly children from grade 2 to grade 5 whose in-group favoritism and out-group spitefulness may have not yet been strongly established. Thus we further propose that the moderating role of in-group and out-group conditions may be evident if elder participants were studied, or if other resource allocation games (e.g., the dictator game) were adopted.

## Limitations, Future Directions, and Strengths

Using a new methodology derived from experimental economics, we objectively measured children’s altruism, and explored the relationships between perceived parenting styles and children’s empathy as well as altruistic behavior. However, this study was not without limitations.

First, participants in this study might be relatively homogenous (from only one province in Eastern China). So the findings should be treated with caution. Future research is needed to replicate these results in more representative samples.

Second, low reliability of two subscales (i.e., maternal and paternal overprotection) may have result in the relationships between variables being inappropriately estimated. Future study may benefit from using more reliable measures of perceived parenting styles.

Third, given that the altruism score derived from each binary-choice dictator game only generated small amount of variances, the influences of perceived parenting styles and children’s empathy on children’s altruistic choices in each game haven’t been analyzed, respectively. That is, we only analyzed the accumulated effects by summing up the scores of these altruistic choices across four economic games. Future research may be more interesting if each binary-choice is treated as outcome and is analyzed with more advanced statistical methods.

Fourth, although children have not interacted with their partner, the presence of the experimenter could also have elicited more prosocial choices to some degree. Future research using multiple data sources and multiple methodologies will provide more robust and valid evidence of altruism.

Fifth, this study did not have fathers’ ratings of their children’s empathy level. It is possible that parents see these behaviors quite differently. Additionally, maternal ratings of empathy are used, so it is not clear if the similar results across the models are simply a function of both using maternal reports of empathy. In future research, children’s empathy could also be assessed via children’s self-report and fathers’ ratings.

Despite these limitations, this study has made several breakthroughs that were stated as follows: First, this study was more effective and creative in using four simple binary-choice dictator games rather than questionnaires or rating scales to measure children’s altruism. The methodology involves experiments without repeated interactions and the partners are anonymous to exclude the expectations of future reciprocity, isolating other-regarding preferences from the strategic behaviors (Fehr et al., 2008, 2013; Bauer et al., 2014). Second, this study tried to explore which kinds of perceived parenting styles are better for children’s empathy and altruistic behavior in Chinese culture. Additionally, this study extends existing research by examining both maternal and paternal parenting styles separately. This may provide more valid systemic perspectives of the influences of both parents’ parenting styles on children’s outcomes. Third, this study confirmed the crucial role of empathy in Chinese children’s prosocial development, as well as the mechanisms that perceived parenting styles influence children’s altruistic behavior regardless the recipients were in-group and out-group members.

## Summary

Prosocial behaviors are beneficial for children’s adjustment and successful development (Eisenberg et al., 2006). But scarce literature has investigated the influences of perceived parenting styles on children’s positive outcomes in Chinese background, especially the mechanisms between them. Employing a combination of experimental and correlational design, this study has filled these research gaps by finding the positive effects of maternal and paternal emotional warmth, as well as the negative effects of maternal and paternal rejection on children’s altruism via children’s empathy. Additionally, this study provides children’s empathy as a mechanism by which perceived parenting styles may heighten children’s altruism both for in-group or out-group conditions. For Chinese parents, more emotional warmth, more support and involvement, and less rejection in parent–child interactions are encouraged. In light of these findings, this study would also suggest that specific attention should be paid to children’s empathy, which is of central importance in fostering children’s prosocial development.

## Author Contributions

QG designed and performed the experiments, as well as contributed research materials and analysis tools. LF analyzed the data and contributed to the writing of the manuscript.

## Conflict of Interest Statement

The authors declare that the research was conducted in the absence of any commercial or financial relationships that could be construed as a potential conflict of interest.
